# Crow deaths as a sentinel surveillance system for West Nile virus in the northeastern United States, 1999.

**DOI:** 10.3201/eid0704.010402

**Published:** 2001

**Authors:** M Eidson, N Komar, F Sorhage, R Nelson, T Talbot, F Mostashari, R McLean, West Nile Virus Avian Mortality Surveillance Group

**Affiliations:** Zoonoses Program, New York State Department of Health, Rm. 621 ESP Corning Tower, Albany, NY 12237, USA. mxe04@health.state.ny.us

## Abstract

In addition to human encephalitis and meningitis cases, the West Nile (WN) virus outbreak in the summer and fall of 1999 in New York State resulted in bird deaths in New York, New Jersey, and Connecticut. From August to December 1999, 295 dead birds were laboratory-confirmed with WN virus infection; 262 (89%) were American Crows (Corvus brachyrhynchos). The New York State Department of Health received reports of 17,339 dead birds, including 5,697 (33%) crows; in Connecticut 1,040 dead crows were reported. Bird deaths were critical in identifying WN virus as the cause of the human outbreak and defining its geographic and temporal limits. If established before a WN virus outbreak, a surveillance system based on bird deaths may provide a sensitive method of detecting WN virus.


### Crow Deaths as a Sentinel Surveillance System for West Nile Virus in the Northeastern United States, 1999

**Millicent Eidson,* Nicholas Komar,† Faye Sorhage,‡ Randall Nelson,§ Tom Talbot,* Farzad Mostashari,¶ Robert McLean,# and the West Nile Virus Avian Mortality Surveillance Group**[1]

*New York State Department of Health, Albany, New York, USA; †Centers for Disease Control and Prevention, Fort Collins, Colorado, USA; ‡New Jersey Department of Health and Senior Services, Trenton, New Jersey, USA; §Connecticut Department of Public Health, Hartford, Connecticut, USA; ¶New York City Department of Health, New York City, New York, USA; #National Wildlife Health Center, Madison, Wisconsin, USA

In addition to human encephalitis and meningitis cases, the West Nile (WN) virus outbreak in the summer and fall of 1999 in New York State resulted in bird deaths in New York, New Jersey, and Connecticut. From August to December 1999, 295 dead birds were laboratory-confirmed with WN virus infection; 262 (89%) were American Crows (*Corvus brachyrhynchos*). The New York State Department of Health received reports of 17,339 dead birds, including 5,697 (33%) crows; in Connecticut 1,040 dead crows were reported. Bird deaths were critical in identifying WN virus as the cause of the human outbreak and defining its geographic and temporal limits. If established before a WN virus outbreak, a surveillance system based on bird deaths may provide a sensitive method of detecting WN virus.

West Nile (WN) virus (family Flaviviridae) causes inapparent infection, mild febrile illness, meningitis, encephalitis, or death in humans and horses in Europe, Africa, Asia, and Australia (1). Wild birds are considered the principal hosts of WN virus, and mosquitoes, particularly *Culex* species, are the primary vector (1). Bird deaths had not been frequently documented in previous human WN virus outbreaks, although infected carcasses of a variety of bird species were found in Israel in 1998 (1,2), and deaths were observed after experimental infection in crows and sparrows (3).

As early as the end of June 1999, an unusual number of dead and dying crows were noted by residents of northern Queens in New York City (NYC). In July, a local veterinarian noted neurologic illness in some birds with unstable gait. Although not then recognized, the earliest cases of human illness due to West Nile virus occurred in this area, beginning in the first week of August (4). After initial evaluation of dead birds by the New York State Department of Environmental Conservation's Wildlife Pathology Unit and the Wildlife Conservation Society, a virus isolated from specimens by the National Wildlife Health Center and the U.S. Department of Agriculture's National Veterinary Services Laboratory was identified as WN virus by the Centers for Disease Control and Prevention (CDC) on September 23 (5). The virus was also recovered by the Connecticut Agricultural Experiment Station in specimens from a Connecticut bird on September 13 (6). A West Nile virus genomic sequence identical to that derived from the bird isolates was then observed in a brain specimen from a human encephalitis case (7).

In response to the initial indications of WN virus in bird specimens, surveillance systems for bird deaths and laboratory testing were established and used in the assessment and control of the outbreak. We reviewed data from systems in New York State, New Jersey, and Connecticut to describe how surveillance of bird deaths was used in 1999 to guide public health action, as well as the advantages and disadvantages of using dead birds as sentinels for West Nile virus in a given geographic area.

### Methods

### Sightings of Ill or Dead Birds

Local health departments were requested to collect and report dead birds to the state health departments of New York

Address for correspondence: Millicent Eidson, Zoonoses Program, New York State Department of Health, Rm. 621 ESP Corning Tower, Albany, NY 12237, USA; fax: 518-473-6590; e-mail: mxe04@health.state.ny.us

[1]Ward Stone, New York State Department of Environmental Conservation; Madhu Anand, Rockland County (NY) Department of Health; Annie Fine, Nancy Jeffery, New York City Department of Health; Ada Huang, Christine Falco, Westchester County (NY) Department of Health; Steve Kopian, Nassau County (NY) Department of Health; Clare Bradley, Suffolk County (NY) Department of Health Services; Kate Schmit, Amy Willsey, Yoichiro Hagiwara, Dennis White, Barbara Wallace, Perry Smith, Hwa-Gan Chang, New York State Department of Health; local and county agencies in New Jersey and Connecticut; Eddy Bresnitz, Colin Campbell, New Jersey Department of Health and Senior Services; Doug Roscoe, New Jersey Department of Environmental Protection; Theodore Andreadis, John Anderson, Charles Vossbrinck, Connecticut Agricultural Experiment Station; James Hadler, Connecticut Department of Public Health; Herbert Van Kruiningen, Antonio Garmendia, Richard French, University of Connecticut; Jenny Dickson, Connecticut Department of Environmental Protection; Lou Sileo, National Wildlife Health Center; and Amy Kerst, Robert Lanciotti, Nicholas A. Panella, Centers for Disease Control and Prevention.

and Connecticut. Sighting reports for ill or dead birds that were not submitted for laboratory testing were not systematically maintained in New Jersey in 1999. Data collected included date of the report, date of death or sighting of the birds, whether the birds were dead or appeared ill, street address where the birds were seen or found, number of birds, and species of birds. Mapping was based on the earliest date provided for the death or sighting. New York State's surveillance data for bird deaths were collected prospectively from September 23, 1999, through November 30, 1999, and retrospectively through May 1, 1999. Connecticut's reporting system was active from September 30, 1999, through November 4, 1999.

In New York State, a geographic information system was used to geocode locations of WN virus-positive birds and to generate maps. Because of incomplete address information, dot-density mapping was used with random placement of the birds within townships for dead crow sightings in New York State and WN virus-positive birds in Connecticut and New Jersey. To assess changes in crow populations, the National Audubon Society's Christmas Bird Count (8), adjusted for party-hours (sum of hours spent counting by each group performing the count), was used.

### Specimen Collection

Recently dead birds with no other obvious causes of death were submitted for testing in all three states. Although initially New York State requested submission only of birds found within 1 mile of each other within 72 hours, that requirement was soon dropped. Connecticut prioritized the submission of birds based on towns with multiple reports of dead birds and then in areas near the towns where WN virus was confirmed. WN virus testing was limited to birds collected from September 13 through October 29, 1999. New Jersey initially accepted all dead bird specimens but later reduced the testing of specimens from several counties where numerous positives had been identified. Mapping was based on the date the dead bird was found.

In their respective states, dead birds were necropsied and specimens were processed for virus testing by the New York State Wildlife Pathology Unit, the New Jersey Department of Health and Senior Services Public Health and Environmental Laboratory, and New Jersey Division of Fish and Wildlife Pathology Laboratory, as well as the Department of Pathobiology at the University of Connecticut.

### Laboratory Testing

Methods for detecting WN virus in avian tissues at CDC have been described (9). Briefly, tissue samples were prepared by macerating approximately 0.5 $cm^3$ of brain tissue in 1.8 mL of BA-1 diluent in a glass TenBroeck tissue grinder (Bellco Glass, Inc., Vineland, NJ). These homogenates were clarified by centrifugation. Virus isolation was attempted in duplicate 100-µL aliquots of the supernatant by Vero plaque assay in 6-well plates. A 75-µL aliquot from each sample was tested by either the traditional or TaqMan reverse-transcriptase-polymerase chain reaction (RT-PCR) assays or both.

In Connecticut, brain tissue was assayed for WN virus as described (6), using cytopathic effect in Vero culture to screen for viruses and specific WN virus RT-PCR for identification. A similar strategy was used at the National Wildlife Health Center, but kidney or spleen suspensions were used in place of brain.

### Results

### Ill or Dead Bird Sightings

New York State received 13,654 reports of 17,339 dead birds from 32 county health departments and from the New York City Department of Health, which represents five boroughs (counties). Dates of death ranged from May 1 to November 30. The predominant species reported was the American Crow (*Corvus brachyrhynchos*) (5,697 sightings, 33%). Before August, there were few retrospective dead crow sightings, and these were confined primarily to the NYC boroughs of Queens and the Bronx and to lower Westchester County. Continued geographic spread of dead crow sightings was noted in August (Figure 1a). Reported sightings peaked in September (Figure 1b), with the largest numbers from NYC and lower Westchester County and wide distribution into Long Island and north along the Hudson River. Although dead crow reports did not dramatically decrease until November, they began to decline in number and density in October (Figure 1c). Later reports were also distributed farther north along the upper Hudson Valley. Most of the dead bird sightings were of single dead birds, rather than clusters of dead birds found together.

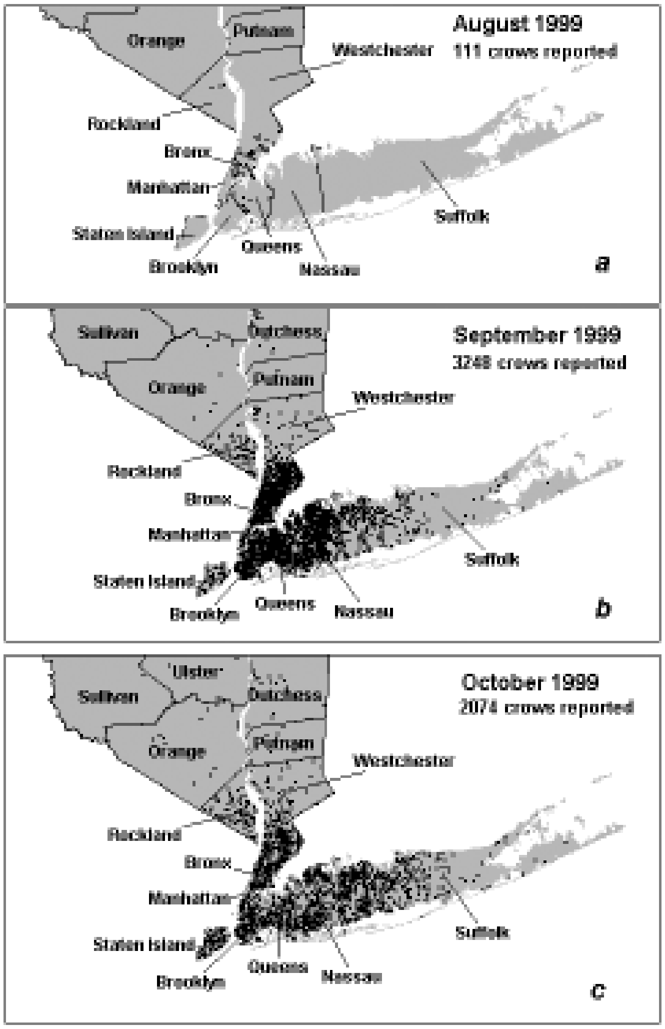

Figure 1. Dead crow sightings, August-October, 1999, New York State.

In Connecticut, the Department of Public Health received reports of dead birds from health departments representing 40 of 169 Connecticut towns. Thirty-five of these 40 towns had reported 1,040 dead crow sightings by the time surveillance ended. The earliest report of a dead crow was in Stratford on September 1, and the latest was in New Fairfield on November 5. The peak number of deaths in a week was 279 during the week of September 26 to October 2, although not all reports included the date of the sighting. Of the 10 towns where more than 10 dead crows were sighted, all were coastal towns, including 8 in Fairfield County and 2 in New Haven County. However, towns in 6 of the 8 Connecticut counties received 1 to 10 reports of dead crows.

### Laboratory Testing

Of 671 dead birds tested, 295 had laboratory-confirmed WN virus infection (142 from New York State, 78 from New Jersey, and 75 from Connecticut). The proportions testing positive were 39% for New York State, 37% for New Jersey, and 77% for Connecticut. WN virus-positive dead birds provided evidence of possible viral activity in four New York State counties, all five NYC boroughs, 16 New Jersey counties, and two Connecticut counties. Viral activity, as indicated by WN virus-positive birds, spread from a central cluster in NYC and adjacent New York State counties in August (Figure 2a) to northeastern New Jersey and southwestern Connecticut in September (Figure 2b). In October, a "central clearing" with fewer WN virus-positive birds in the NYC area was evident (Figure 2c), while a wider distribution of infected birds was seen in southern New Jersey. In Connecticut, where testing was primarily in towns near areas with confirmed WN virus-infected birds, fewer WN virus-positive birds were identified in October than in earlier months.

Two hundred sixty-two (89%) of the WN virus-positive dead birds were American Crows. However, WN virus was isolated from dead birds of 19 other species, including the Fish Crow (*C. ossifragus*, 7), Chilean Flamingo (*Phoenicopterus chilensis*, 4), Blue Jay (*Cyanocitta cristata*, 4), Red-tailed Hawk (*Buteo jamaicensis*, 2), Mallard (*Anas platyrhynchos*, 2), and one each of the following species: Rock Dove (*Columba livia*), Belted Kingfisher (*Ceryle alcyon*), Laughing Gull (*Larus atricilla*), Herring Gull (*L. argentatus*), Black-crowned Night Heron (*Nycticorax nycticorax*), Sandhill Crane (*Grus canadensis*), Guanay Cormorant (*Phalacrocorax bougainvillea*), Blyth's Tragopan (*Tragopan blythi*), Bald Eagle (*Haliaeetus leucocephalus*), American Kestrel (*Falco sparverius*), Broad-winged Hawk (*Buteo platypterus*), Cooper's Hawk (*Accipiter cooperii*), Merlin (*Falco columbarius*), and American Robin (*Turdus migratorius*). The noncorvid species were primarily from New York State, except for a Cooper's Hawk and Sandhill Crane reported from Connecticut and a Red-tailed Hawk and Merlin reported from New Jersey.

The earliest collection dates for WN virus-positive birds were August 2-9 in Nassau County, New York (Figure 3), and the latest collection date was November 15, from Rockland

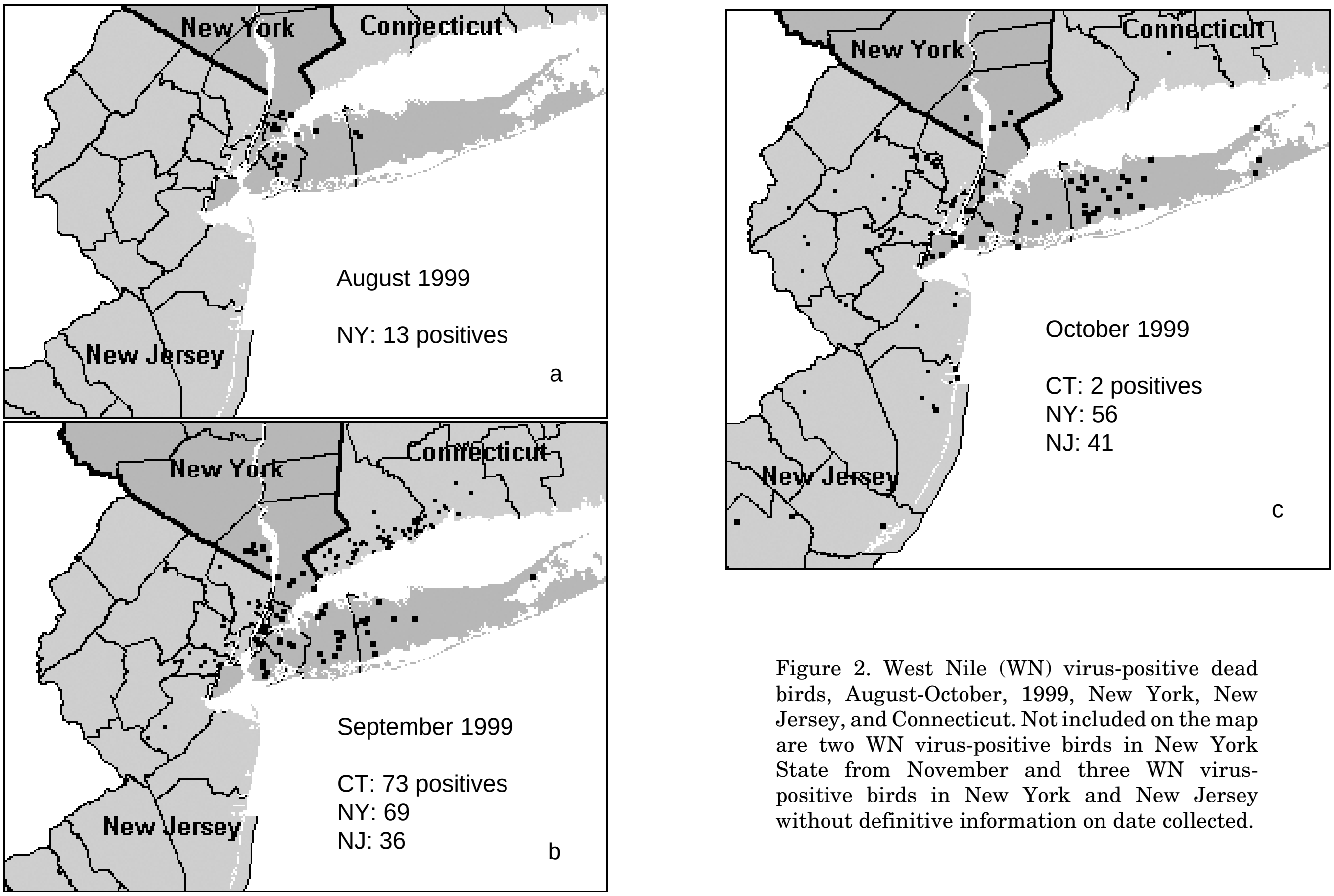

Figure 2. West Nile (WN) virus-positive dead birds, August-October, 1999, New York, New Jersey, and Connecticut. Not included on the map are two WN virus-positive birds in New York State from November and three WN virus-positive birds in New York and New Jersey without definitive information on date collected.

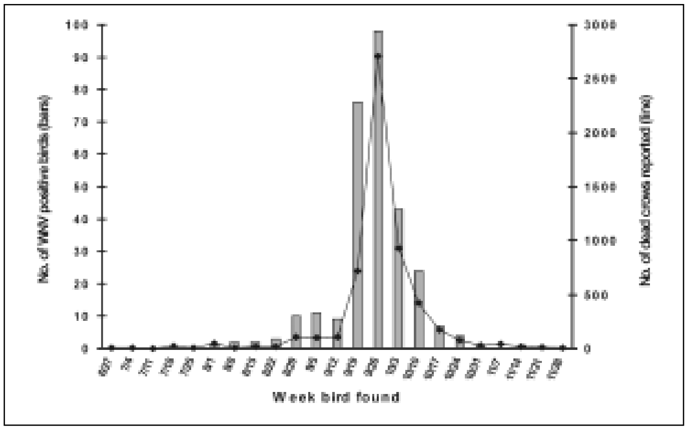

Figure 3. Number of dead crow sightings in New York State and number of West Nile (WN) virus-positive birds in New York State, New Jersey, and Connecticut, by week, June 27-November 30, 1999. Not included are three WN virus-positive birds in New York and New Jersey without definitive information on date collected.

County, New York. The peak in collections of WN virus-positive birds, as well as reports of dead crow sightings in New York State and Connecticut, occurred during the week of September 26, immediately after the first press release announcement that WN virus had been detected in dead birds.

Analysis of the National Audubon Society's Christmas Bird Count data, adjusted for party-hour (8), indicated a decrease in the number of crows sighted in 1999 (after the WN virus outbreak) compared with 1998, with the largest decreases in the NYC WN virus epicenter boroughs of Queens (69%) and the Bronx (65%) (Figure 4). Geographic areas at the periphery of the outbreak in 1999, including Rockland County, Staten Island, and the eastern tip of Suffolk County, had increases in crow sightings in 1999 compared with 1998.

Retrospective testing found no WN virus-positive birds among six archived specimens found dead in the New York City region from May 27 to August 16, 1998 (including two American Crows) or among three specimens collected in April 1999 in the same region.

### Conclusion

Although inapparent avian infections were known to occur during WN virus outbreaks, along with occasional avian illnesses and deaths (2,10), the WN virus outbreak in the northeastern United States in 1999 is the first with a recognized substantial avian mortality rate (1).

Interpretation of the results of this surveillance system in 1999 in the Northeast and conclusions about its possible future value as a sentinel for WN virus have several limitations. First, bird death cannot be adequately investigated over wide areas without recognition of its importance by the public and by local and state agencies in those areas. Routine mechanisms were already in place at the local, state, and federal levels to investigate bird die-offs, and wildlife, zoologic, health, and agricultural agencies played a critical role in determining the presence of WN virus in this hemisphere. However, public knowledge of the WN virus outbreak did result in a peak in the number of reported dead birds, occurring immediately after the first press announcement of WN virus. Thus, public awareness of the need to report animal deaths is key to using ill or dead wild animals as sentinels for detection of zoonotic pathogens.

Another limitation is that media coverage was more intense in areas close to NYC and where the first WN

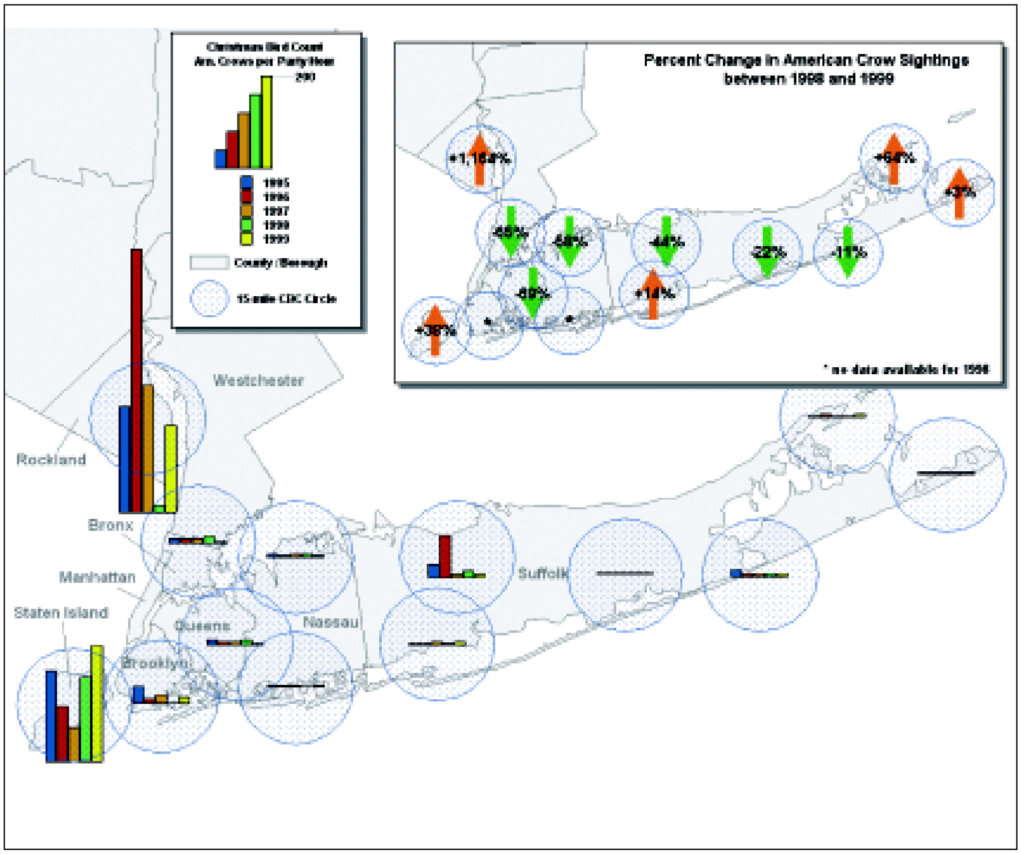

Figure 4. Christmas bird count, number of American Crows reported, adjusted for party-hours, 1995-1999, New York City area (8).

virus-positive birds were found, which may have influenced public awareness of the surveillance system and led to underreporting of dead birds in areas with less media coverage. An active system of surveillance for bird deaths may be necessary to supplement passive reporting systems in areas without strong media coverage and public awareness about the need to report dead birds.

The process of obtaining birds for necropsy and performing laboratory analyses proved to be time-consuming and labor-intensive, so that testing had to be prioritized and limited. Thus, in addition to potential variability in the quality of the reporting of dead bird sightings, additional problems in interpreting data on positive birds may result from differing decision processes and procedures for collection and submission of birds for testing across county and state lines.

Drawing any definitive conclusions about the decreases in 1999 crow counts seen by the National Audubon Society in the epicenter of the outbreak is problematic. The percentage of reductions in the numbers of American Crows seen is based on small numbers of birds per party-hour. In addition, the counts may be influenced by factors such as crow migration in the fall and changes in the number and skill of bird survey participants from year to year.

An additional limitation of the possible usefulness of bird deaths as a sentinel for WN virus is the difference between the outbreaks in humans and birds in the Northeast in 1999. The geographic distribution of positive birds was much greater than that of human cases. No human cases were reported from Connecticut and New Jersey despite positive dead birds in 2 and 16 counties, respectively, and no human cases were reported from one NYC borough and two New York State counties with positive birds (11). Some of the positive birds may not have provided indication of viral activity and risk to humans in the counties where they were found because they could have been infected elsewhere and flown to a different county before their death.

A final limitation is that WN virus was confirmed in humans and birds at the same time, in late September 1999, for humans and birds with onset of illness in early August (11). Therefore, analysis of avian mortality in 1999 cannot definitively determine whether a prospectively established surveillance system could have provided an early warning for detecting human cases in 1999. However, an increase in dead crow sightings in June in 1999 was one indication that such surveillance could have provided an early sign of possible viral activity.

Despite these limitations, the pattern of crow death reports corresponded with the pattern of WN virus-positive birds, and a clear geographic spread for virus detection can be discerned by examining the maps of dead crow sightings and WN virus-positive birds. A laboratory study of Hooded Crows (*Corvus corone sardonius*) in Egypt infected with WN virus by mosquito bites found that the birds died 1-7 days (median 4 days) after being bitten (3). Thus, dead crows may provide a sensitive indicator of continuing WN virus transmission in an area even after WN virus isolations in mosquitoes or cases in humans or other animals are no longer reported, for example, in the autumn.

Although most of the WN virus-positive dead birds in this study were crows, we emphasize that the mortality impact of WN virus on other bird species has not been adequately studied. This report indicates that 20 species of birds were found to be WN virus-positive during 1999, in spite of the fact that surveillance efforts focused on crows. Eight of these 20 positive species represented captive birds from zoological collections. Natural WN virus infection in seven of these species plus an additional three species of captive birds infected in 1999 have been described (12). However, although 11 of the 23 species of birds now known to have been infected with WN virus in the United States in 1999 were captive when infected, 19 are also wild resident bird species. Thus, WN virus clearly represents a threat to both zoo collections and the native avifauna of North America, in addition to people and horses. As such, in 1999 the National Wildlife Health Center and CDC established ongoing dead and live bird surveillance systems along the East Coast of the United States, first on federal and state natural resource lands and then in conjunction with state public health and animal health agencies.

In summary, the WN virus outbreak in the northeastern United States in late summer and early fall 1999 represented the first introduction of WN virus into the Western Hemisphere. This WN virus outbreak was remarkable in the large numbers of observed crow fatalities and the importance of surveillance for monitoring the outbreak and making public health surveillance and disease control decisions. Establishment of surveillance for bird deaths before possible introduction of the virus in an area, along with additional analyses to identify correlates with human cases, will be required to provide more accurate and timely projections of the likelihood of human cases.

### Acknowledgments

The authors thank the following for their assistance in surveillance of bird deaths: Stephanie Anderson, Gary Archambeault, Geraldine Bunn, Scott Campbell, Kathy Carlton, Haiyan Chen, Sue Cofer, Bruce Cropp, Tomas Foral, David Graham, Gail Hein, Kim Holmes, Brian Hunderfund, Geraldine Johnson, Mary Keegan, Geoff Krukar, Claudia Lee, Jennifer Maratea, Kathryn McLaughlin, Craig Morin, Giovanni Rompato, Amy Schrom, Steve Segore, Kevin Sullivan, Charles Trimarchi, Megan Tucker, Randi Walker, and Al Zlomek.

Dr. Eidson is State Public Health Veterinarian and Director of the Zoonoses Program, New York State Department of Health. In addition, she is an associate professor in the Department of Epidemiology, State University of New York School of Public Health, and a diplomate of the American College of Veterinary Preventive Medicine. Her research focuses on rabies and West Nile virus.

### References

1. Komar N. West Nile viral encephalitis. Rev Sci Technol 2000;19:166-76.
2. Malkinson M, Banet C, Weisman J, Pokamonski S, King R. West Nile fever: recent evidence for inter-continental dispersion of the virus by migratory birds. Proceedings of the 11th International Congress of Virology; 1999 August 9-13; Sydney, Australia. Sydney: International Union of Microbiological Societies; 1999. p. 56.
3. Work TH, Hurlbut HS, Taylor RM. Indigenous wild birds of the Nile Delta as potential West Nile virus circulating reservoirs. Am J Trop Med Hyg 1955;4:872-88.
4. Centers for Disease Control and Prevention. Outbreak of West Nile-like viral encephalitis—New York, 1999. MMWR Morb Mortal Wkly Rep 1999;48:845-9.
5. Lanciotti RS, Roehrig JT, Deubel V, Smith J, Parker M, Steele K, et al. Origin of the West Nile Virus responsible for an outbreak of encephalitis in the northeastern United States. Science 1999;286:2333-7.

6. Anderson JF, Andreadis TG, Vossbrinck CR, Tirrell S, Wakem EM, French RA, et al. Isolation of West Nile virus from mosquitoes, crows and a Cooper's hawk in Connecticut. Science 1999;286:2331-3.
7. Jia XY, Briese T, Jordan I, Rambaut A, Chi HC, Mackenzie JS, et al. Genetic analysis of West Nile New York 1999 encephalitis virus. Lancet 1999;354:1971-2.
8. National Audubon Society Christmas Bird Count. Available from: URL:http://www.birdsource.org/cbc
9. Lanciotti RS, Kerst AJ, Nasci RS, Godsey MS, Mitchell CJ, Savage HM, et al. Rapid detection of West Nile virus from human clinical specimens, field-collected mosquitoes, and avian samples by a TaqMan reverse transcriptase-PCR assay. J Clin Microbiol 2000;38:4066-7.
10. Taylor RM, Work TH, Hurlbut HS, Rizk F. A study of the ecology of West Nile virus in Egypt. Am J Trop Med Hyg 1956;5:579-620.
11. Nash D, Mostashari F, Fine A, Miller J, O'Leary D, Murray K, et al. Outbreak of West Nile virus infection, New York City area, 1999. N Engl J Med. In press 2001.
12. Steele KE, Linn MJ, Schoepp RJ, Komar N, Geisbert TW, Manduca RM, et al. Pathology of fatal West Nile virus infections in native and exotic birds during the 1999 outbreak in New York City. J Vet Pathol 2000;37:208-24.

## References

[PDF_00428] Anderson J. F., Andreadis T. G., Vossbrinck C. R., Tirrell S., Wakem E. M., French R. A., Garmendia A. E., Van Kruiningen H. J. (1999). Isolation of West Nile virus from mosquitoes, crows, and a Cooper's hawk in Connecticut.. Science.

[PDF_00419] Centers for Disease Control and Prevention (CDC) (1999). Outbreak of West Nile-like viral encephalitis--New York, 1999.. MMWR Morb Mortal Wkly Rep.

[PDF_00440] HURLBUT H. S., RIZK F., TAYLOR R. M., WORK T. H. (1956). A study of the ecology of West Nile virus in Egypt.. Am J Trop Med Hyg.

[PDF_00431] Jia X. Y., Briese T., Jordan I., Rambaut A., Chi H. C., Mackenzie J. S., Hall R. A., Scherret J., Lipkin W. I. (1999). Genetic analysis of West Nile New York 1999 encephalitis virus.. Lancet.

[PDF_00409] Komar N. (2000). West Nile viral encephalitis.. Rev Sci Tech.

[PDF_00436] Lanciotti R. S., Kerst A. J., Nasci R. S., Godsey M. S., Mitchell C. J., Savage H. M., Komar N., Panella N. A., Allen B. C., Volpe K. E. (2000). Rapid detection of west nile virus from human clinical specimens, field-collected mosquitoes, and avian samples by a TaqMan reverse transcriptase-PCR assay.. J Clin Microbiol.

[PDF_00422] Lanciotti R. S., Roehrig J. T., Deubel V., Smith J., Parker M., Steele K., Crise B., Volpe K. E., Crabtree M. B., Scherret J. H. (1999). Origin of the West Nile virus responsible for an outbreak of encephalitis in the northeastern United States.. Science.

[PDF_00445] Steele K. E., Linn M. J., Schoepp R. J., Komar N., Geisbert T. W., Manduca R. M., Calle P. P., Raphael B. L., Clippinger T. L., Larsen T. (2000). Pathology of fatal West Nile virus infections in native and exotic birds during the 1999 outbreak in New York City, New York.. Vet Pathol.

[PDF_00416] WORK T. H., HURLBUT H. S., TAYLOR R. M. (1955). Indigenous wild birds of the Nile Delta as potential West Nile virus circulating reservoirs.. Am J Trop Med Hyg.

